# Inequity and benefit incidence analysis in healthcare use among Syrian refugees in Egypt

**DOI:** 10.1186/s13031-021-00416-y

**Published:** 2021-11-02

**Authors:** Hani Fares, Jaume Puig-Junoy

**Affiliations:** 1 United Nations High Commissioner for Refugees (UNHCR), 1202 Geneva, Switzerland; 2 Universitat Pompeu Fabra-Barcelona School of Management (UPF-BSM), C. Balmes 132-134, 08007 Barcelona, Catalonia Spain

**Keywords:** Refugees’ health, Socioeconomic inequalities, Inequity, Horizontal inequity, Syrian refugees

## Abstract

**Background:**

The Syrian conflict has created the worst humanitarian refugee crisis of our time, with the largest number of people displaced. Many have sought refuge in Egypt, where they are provided with the same access to healthcare services as Egyptian citizens. Nevertheless, in addition to the existing shortcomings of the Egyptian health system, many obstacles specifically limit refugees’ access to healthcare. This study looks to assess equity across levels of care after observing services utilization among the Syrian refugees, and look at the humanitarian dilemma when facing resource allocation and the protection of the most vulnerable.

**Methods:**

A cross‐sectional survey was used and collected information related to access and utilization of outpatient and inpatient health services by Syrian refugees living in Egypt. We used concentration index (CI), horizontal inequity (HI) and benefit incidence analysis (BIA) to measure the inequity in the use of healthcare services and distribution of funding. We decomposed inequalities in utilization, using a linear approximation of a probit model to measure the contribution of need, non-need and consumption influential factors.

**Results:**

We found pro-rich inequality and horizontal inequity in the probability of refugees’ outpatient and inpatient health services utilization. Overall, poorer population groups have greater healthcare needs, while richer groups use the services more extensively. Decomposition analysis showed that the main contributor to inequality is socioeconomic status, with other elements such as large families, the presence of chronic disease and duration of asylum in Egypt further contributing to inequality. Benefit incidence analysis showed that the net benefit distribution of subsidies of UNHCR for outpatient and inpatient care is also pro-rich, after accounting for out-of-pocket expenditures.

**Conclusion:**

Our results show that without equitable subsidies, poor refugees cannot afford healthcare services. To tackle health inequities, UNHCR and organisations will need to adapt programmes to address the social determinants of health, through interventions within many sectors. Our findings contribute to assessments of different levels of accessibility to healthcare services and uncover related sources of inequities that require further attention and advocacy by policymakers.

## Background

A refugee is someone who ‘owing to a well-founded fear of being persecuted for reasons of race, religion, nationality, membership of a particular social group or political opinion, is outside the country of his nationality, and is unable to, or—owing to such fear—is unwilling to avail himself of the protection of that country’ [1]. The Syrian conflict has created the worst humanitarian refugee crisis of our time, with the largest number of people displaced, many of whom have found refuge in Egypt. As of August 2017, the country counted 131,504 Syrian refugees registered with the United Nations High Commissioner for Refugees (UNHCR), in addition to 105,885 refugees from other nationalities [2].

The socio-economic situation of refugees and asylum seekers mirror those of the urban poor in Egypt as they face multiple obstacles including dramatic price rises and inflation related to the ongoing economic reforms in the country, scarce employment opportunities, and a general deterioration of the security environment due to political instability in the era post-revolution. Many refugees are subsequently highly vulnerable, according to the UNHCR 2017 vulnerability assessment, 40% of refugees living in Cairo are considered poor, 20% extremely poor [3, 4].

The healthcare system in Egypt is quite complex with many public entities involved in the management, El-Zanaty and Way [5] financing and provision of care and heavy reliance of the Egyptian citizens on out-of-pocket (OOP) health payments causes financial burdens for households [6, 7].

Syrian refugees have access to the same primary healthcare services as Egyptian citizens, via public health clinics. Nevertheless, many obstacles limit refugees’ healthcare access—for example, insufficient financial means and out-of-pocket payments, shortage of drugs and investigations, insensitive encounters with national service providers, and inadequate information on types and place of availability of health services [4, 8].

Moreover, refugees’ face further health challenges, caused by epidemiologic and demographic dynamics, such as the high burden of non-communicable diseases due to ageing populations and unhealthy lifestyles, combined with a prevalence of communicable diseases related to poor hygiene and lack of access to basic health services [9, 10], resulting in the need for necessary healthcare use being likely to increase [11].

In light of the competing needs of the refugees (health, education, food …), and the continuous reduction in international funding, UNHCR has adopted a mixed public health approach, which prioritizes affordable and essential primary health care through public facilities, while chronic diseases, reproductive health and mental health care services are provided through dedicated UNHCR supported facilities and affiliated hospitals for life-saving secondary and tertiary care. Accordingly, the packages of health assistance offered to the refugees and asylum seekers in Egypt have raised ethical and operational concerns, specifically on how services could reach the most vulnerable refugees.

The World Health Organisation has stated that the progressive realization of the right to health involves a concerted and sustained effort to improve health across all populations and reduce inequities in the enjoyment of health; and has defined Equity as ‘the absence of avoidable or remediable differences among groups of people, whether those groups are defined socially, economically, demographically, or geographically’ [12]. However, this definition does not include the financing of healthcare payments which is an integral part of a health system [13, 14]. Therefore, equity in healthcare shall encompass two main principles, fair allocation of resources and equitable access to healthcare services.

Several studies have shown that, in both developed and developing countries, when individuals’ health needs are not equitably addressed [15, 16], this results in unmet health needs, inadequate healthcare, inequitable health outcomes, and increased costs [17–19]. Moreover, the lack of health insurance and the weak purchasing power among poor refugees may result in less utilization of healthcare services despite their greater need [20, 21].

Public health equity issues facing the humanitarian community have been previously outlined into issues of resource allocation and decision-making [22–25]. Ensuring greater equity in the use of needed health services and financial protection when accessing those services are fundamental policy objectives for countries, and organisations like UNHCR, that seek to protect the people in concern and the most in need among them [15, 26]. While the research on equity in healthcare has been mainly concerned with four broad sets of outcomes [27, 28]: (1) healthcare utilization according to health needs irrespective of socio-economic status, ability to pay, social or personal background (horizontal equity); (2) subsidies received using services; (3) payments people make for healthcare (out-of-pocket payments, insurance premiums); and (4) health status.

In this study, we look closer at the humanitarian dilemma when facing the distribution of funding and the protection of the most vulnerable when accessing healthcare. We aim then, to assess inequity across levels of care, after observing services utilization among the Syrian refugees and analysing the allocation of UNHCR healthcare subsidies from an adequacy perspective. To achieve these purposes, we develop three specific objectives: (i) To determine inequalities in the distribution of healthcare utilization across socioeconomic groups and demographic characteristics of the individuals and households; (ii) To assess the level of inequities in healthcare use and contributing factors by measuring horizontal inequity (HI); and, (iii) To determine whether the contribution of UNHCR subsidies are appropriately distributed across refugees’ socioeconomic groups, and assess the extent to which different groups benefit from those subsidies through their use of health services.

This paper provides a better understanding of the overall performance of the refugee health program in Egypt and contributes to the development of appropriate and equitable policies. To our knowledge, no previous studies have analysed the socio-economic inequity in the use of healthcare services within the refugee’s population.

## Methods

### Study design

A cross‐sectional survey of Syrian refugees in Egypt was conducted in September 2017 to characterize health‐seeking behaviours and barriers related to accessing health. A representative random sample of Syrian households registered with UNHCR Egypt was contacted by telephone.

This Health Access and Utilization Survey (HAUS) is a monitoring tool for UNHCR to collect information on access and utilization of healthcare services in many countries [29].

### Data collection and questionnaire

A stratified systematic random sampling was used to attain a nationally representative sample of Syrian refugee households, which are registered with UNHCR and have a phone number listed in the UNHCR database. Stratification was based on Syrian refugees’ geographical distribution in Egypt’s six governorates (Giza, Greater Cairo, Alexandria, Damietta, Sharkia and Qalyubia).

The sample size was estimated at 384 refugee households assuming a precision of 10% and using a level of significance of 0.05 [30]. However, to account for the non-response that was experienced in previous surveys, 914 Syrian households were contacted, and a total of 507 household’s responses were retained and included in our analysis. Households that have been contacted with no response after 3 contact attempts, or with invalid phone numbers and households which didn’t agree to participate in the survey have been excluded from the study. To protect the anonymity of respondents, no information was recorded that could be used to identify the household or individuals and verbal consent was obtained and recorded from all respondents.

The questionnaire has been accustomed, with partner agencies, to set out health sector priorities in Egypt and focus on health services utilization, access to care, barriers to care-seeking, household expenditures, and non-communicable chronic disease (NCD). The questionnaire was translated into Arabic.

Household heads and primary caretakers of children were prioritized to answer questions on behalf of the household and its members. Household members were defined as people who share a dwelling space and share meals, regardless of biological relation. To protect the anonymity of respondents, no information was recorded that could be used to identify the household or individuals and verbal consent was obtained from all respondents.

The first part of the questionnaire included information about demographic characteristics and health status of the household head and each household member. The characteristics of 1872 individuals included age, gender and physical conditions (presence of disability and the presence of one or more chronic disease); the predefined list of NCD included hypertension; diabetes; musculoskeletal disorders; respiratory diseases or asthma; cardiovascular diseases; digestives disorders; epilepsy and mental illness. Data was also collected on household income, consumption of goods including food, rent, transportation, spending on education, and out-of-pocket health expenditures during that month [31]. All expenditures were noted in Egyptian Pound (EGP) and converted into USD using the 2017 exchange rate (1 USD = 17.6 EGP). The second part of the questionnaire collected information on the utilization of health services by each individual based on outpatient use and inpatient admission and defined in four variables: (1) The probability of public outpatient visit was calculated from the question “In the past month have you visited a public health centre (Family Medicine centre, Maternal, Child health centre), a UNHCR Supported clinic or NGO clinic, NGO/charity clinic or a public hospital, for outpatient care consultation?”; (2) The probability of private outpatient visit was calculated from the question “In the past month have you visited a private health centre (Family Medicine centre, Maternal, Child Health centre) or a private hospital, for outpatient care consultation?”; (3) The probability of public inpatient visits was calculated from the question “Have you received been hospitalised during the last 12 months in public or UNHCR supported hospital?”; (4) The probability of private inpatient visits was calculated from the question “Have you received been hospitalised during the last 12 months in a private facility?” In our dataset, all four probabilities may only be equal to one (at least one consultation within the month or at least one hospital stay within the year) or zero.

Data management and descriptive analysis were conducted using SPSS 24.0, while inequity analysis and the Benefit Incidence Analysis (BIA) were conducted using the ADePT software tool developed by the World Bank [32].

### Statistical analysis

For this study, we define inequality as any significant differences in healthcare use between sub-populations, and inequity as the part of inequality that is considered unjustified, where factors correlated with healthcare are considered unfair due to the inability to access equal care based on need, regardless of socioeconomic status [33]. To measure inequity, inequality in the utilization of healthcare will be standardized for differences in justified need (age, gender, NCD, and disability) and unjustified non-need (socioeconomics determinants).

#### Concentration index measuring socioeconomic-related inequality in healthcare use

We adopt the methodology described by O’Donnell et al. [27] or the measurement of the concentration indexes and the decomposition of inequalities. This method has previously been used for equity analysis in several developing countries [34–38].

For developing countries, arguments were made preferring consumption, based on both conceptual and practical considerations [39], Income is received only intermittently, whereas consumption can be “smoothed” over time. In this study, given the poor reliability and volatility of reported income of refugees in the informal market, per-capita household consumption was used as a proxy measure of income or living standards [38, 40, 41]. Accordingly, we have collected data on expenditures for four main classes (i) food, (ii) non-food, non-durable items (as hygiene, clothes, transportation) (iii) consumer durables (as for rent and utilities), and (iv) housing [42, 43], in addition to the expenditure on education and health and total expenditures.

To reduce the impact of household economies of scale, it was adjusted to adult equivalence, as follows:

$${\mathbf{Eqconsumption}} = {\mathbf{household}}\;{\mathbf{consumption}}/({\mathbf{family}}\;{\mathbf{size}})^{{{\mathbf{0}}.{\mathbf{56}}}}$$

The value of the adult equivalent consumption has *been* estimated from previous studies based on 59 countries’ household survey data, and it equals 0.56 [44]. Adult equivalent consumption (*Eqconsumption*) was also used as the ranking variable for income status of the individual.

The concentration index (CI) was used [27, 41, 45] to assess socioeconomic inequality in utilization by type of health services as we present the analysis of health services utilization by equivalised per capita consumption quintile. The concentration index is defined as twice the area between the concentration curve and the line of equality (the 45-degree line). CI for the actual utilization of healthcare services ranges between − 1 and 1. When the CI takes a negative value, it indicates a disproportionate concentration of healthcare use among the poor. The CI is calculated using the covariance between healthcare use and the fractional rank of the individual sorted by consumption status:

1 $$CI = \frac{2}{y} + Cov_{w} (y_{i} ,{\text{R}}_{i} )$$

where *y*_*i*_, is the binary variable of whether the *i*th person had used public, private outpatient service in the previous month, or the previous year for inpatient, *y* stands for the mean of actual healthcare use, *R*_*i*_ denotes the fractional rank of the *i*th individual in the consumption status distribution, with i = 1 for the poorest and i = N for the richest; and, *Cov*_*w*_ is the covariance between the healthcare use variable and fractional rank of the individual sorted by the consumption distribution, with sampling probability weights [30, 46–48]. In a cross-sectional survey, we may find differential response rates, then the completed sample may not be fully representative of the refugees from which it is selected. Weights re-adjust the distribution of the sample to reflect the distribution of the refugee population more accurately [31]. Governorate population was used to construct a sample weighting variable that enabled us to generalise the results at the distribution of the refugees at the national level.

Given that our health utilization variables were binary (1, 0), the bounds of the CI are not − 1 and 1 but depend on the mean of the variable [49–51]. A Wagstaff normalisation process that ensures that the CI is quantified in the range − 1 to 1, for any given mean of the health utilization variables is applied by multiplying the calculated CI by (1/(1 − µ) [49].

Recently, there has been a debate regarding the appropriate normalization process between Wagstaff, stated as CIW = CI * (1 − μ) [49, 50] and Erreygers’s correction of the CI (stated as (4μ/b − a) * CI, where, a and b are upper and lower limits of the health variable, CI is the standard concentration index, and μ is the mean of the health variable) [51, 52]. The key difference between these two propositions is that the first one is a relative measure of inequality, while the second one is an absolute measure of inequality, but Kjellsson and Gerdtham proposed that the choice depends on the researchers’ perspective about relative or absolute inequity [53].

As we focus on this paper on the extent of inequity in healthcare use, we, therefore, adopt the normalization process proposed by Wagstaff for all the CI analysis, however, we introduce Erreygers’s correction beside Wagstaff’s normalisation for the total healthcare utilization in Table 3.

#### Decomposition of inequality

The CI measures the degree of inequality in healthcare use by consumption, however, our interest also lies in measuring the extent of inequity on healthcare use. Horizontal equity here is defined as “equal treatment for equal need, irrespective of other characteristics such as income, race, place of residence, etc.” [28]. Then, to explain inequity, we use the decomposition approach, which partitions the factors contributing to inequity in healthcare use. Following O’Donnell et al.[27], if a healthcare use is specified as a linear function of determinants, then the CI can be decomposed into the contribution of each determinant, computed as the product of the healthcare use variable’s elasticity with respect to the determinant and the latter’s CI. The CI for need-standardized use is exactly equal to that which is obtained by subtracting the contributions of all need variables from the unstandardized CI [27, 34].

To decompose total inequality, we constructed two additional healthcare utilization variables: (1) The total healthcare utilization for outpatient services was recorded, if the person reported at least a consultation in either public or private outpatient facility in the last month; and, (2) The total healthcare utilization for inpatient services was recorded if the person reported one hospitalisation in either public or private in the last year. The total healthcare utilization is equal to 1 if the person reported at least one consultation during the last month or a hospital stay during the last year, either in public or private services and zero otherwise.

The independent variables in the regression model were classified into three groups: (1) need variables, (2) consumption, and (3) other non-need variables to assess the extent to which each of these variables contributes to any inequality in healthcare utilization.

Several studies have shown that how healthcare utilization could be influenced by factors such as educational level [54, 55], socioeconomic status [56], presence of chronic illness [36, 57, 58], and family support [59, 60]. Based on this evidence, we defined healthcare need in terms of the patient’s health and disease status, as measured by the presence of NCDs, and disability. In addition, since healthcare need is often gender and age-specific, we adopted gender and age as proxy need-measures of healthcare. The selection of other non-need factors, besides consumption, included Urban, size of the household, duration since the arrival to the country, Governorates, employment, level of education of the head of the household, and the knowledge of health services availability [58, 61].

We used a linear approximation of a probit model with the partial effects evaluated at means [41, 46]. Taking healthcare use as the dependent variable, the empirical model is expressed via the following linear model:

2 $$y_{i} = \alpha^{m} + \sum\limits_{i} {\beta_{j}^{m} x_{ij} + \sum\limits_{k} {\gamma_{k}^{m} z_{ik} + \varepsilon_{i} } }$$

where *i* denotes the *i*th individual, *x*_*ij*_ refers to the *j*th need factor of the *i*th individual, z_ik_ is the *k*th other non-need and consumption factor, and $\alpha^{m}$ is the intercept; $\beta_{j}^{m}$ and $\gamma_{k}^{m}$ are the marginal effects, *dy*/*dx*_*j*_ and *dy*/*dz*_*k*_, of each need (*x*) and non-need/consumption (*z*) factor evaluated at sample means; and $\varepsilon_{i}$ is the implied error term, which includes approximation errors [46].

Given that in Eq. (2), the concentration index (CI) is linearly additive, the decomposition result could be applied, and then the CI for y is written as [27]:

3 $$CI = \sum\limits_{j} {\left( {\beta_{i}^{m} \overline{x}_{j} /\mu } \right)C_{j} + \sum\limits_{k} {\left( {\gamma_{k}^{m} \overline{z}_{k} /\mu } \right)C_{k} + GC_{s} /\mu } }$$

where *μ* is the mean of *y*, *C*_*j*_ and *C*_*k*_ are the CI of *x*_*j*_ and *z*_*k*_ respectively and calculated similarly to Eq. (1). In the last term, GC_ε_ (residual) is the generalized CI for the error term ε [30]. The residuals reflect the part of the CI that is not due to the factors included in the analysis.

The elasticity term $\left( {\beta_{i}^{m} \overline{x}_{j} /\mu } \right)$ indicates the impact of each determinant on the desired health outcome, and the products ($\left( {\beta_{i}^{m} \overline{x}_{j} /\mu } \right)C_{j}$ and $\left( {\gamma_{k}^{m} \overline{z}_{k} /\mu } \right)C_{k}$ are the contribution of a need factor *j* and a non-need and consumption factor *k* to the actual concentration index, respectively. The positive (negative) partial contribution indicates that the determinant increases (decreases) the total inequality in healthcare utilization, with positive (negative) percentages referring to increases (decreases) in percentages.

We estimated a regression model of healthcare use as a function of both need and non-need variables to predict the need for healthcare avoiding omitted variable bias [27].

### Horizontal inequity (HI)

To measure inequity, inequality in the utilization of healthcare has been standardized for differences in need. After standardization, any residual inequality in utilization is interpreted as horizontal inequity, which could be pro-rich or pro-poor. The decomposition method allows horizontal inequity in utilization to be measured and explained in a convenient way [28]. Equation (3) could be used to estimate need-standardised use. Then, being $\hat{y}_{i}^{X}$ the need predicted values of the healthcare use indicator, it could be obtained as [27]:

4 $$\hat{y}_{i}^{X} = \hat{\alpha } + \sum\limits_{j} {\hat{\beta }_{j} } x_{ji} + \sum\limits_{k} {\hat{\gamma }_{k} \overline{z}_{k} }$$

And, then we calculated the need-standardised use as:

5 $$\check{y}_{i}^{IS} = y_{i} - \hat{y}_{i}^{X} + \overline{\hat{y}}$$

where ($\overline{\hat{y}}$) is the mean of the predictions from Eq. (2) with all variables at actual values. The mean of the predicted value ($\overline{\hat{y}}$) was added to indirectly standardise values to ensure that the mean of the actual use equals the mean of the need-standardised use. Finally, consumption-related HI was then measured by estimating the CI of the need-standardised use [27, 34].

Like the CI, the HI index takes the value between − 1 and + 1 with a zero indicating no inequity in healthcare use. A negative (positive) and significant HI estimate indicates inequity is pro-poor (pro-rich) or healthcare use is more concentrated among the poor (rich) given the same level of health need among the individuals of the consumption distribution. The higher the absolute value of the HI index, the greater the degree of inequity. And, since healthcare use variables were binary, we also corrected the HI indices applying the Wagstaff normalisation [49].

#### Benefit incidence analysis (BIA)

BIA is a method that has been applied in the literature to measure the extent of equity in public subsidy distribution across socio-economic classes [62–68]. We use the BIA method to describe the distribution of UNHCR spending across individuals ranked by their living standards [27, 32, 66] and to identify to which extent the UNHCR subsidy is pro-poor or pro-rich to the health sector. This method involves four steps [66]: (1) measuring the living standards or socio-economic status of the population; (2) estimating the unit cost attached to each service utilised (visit for outpatient and at least 1-day hospitalisation for inpatient use); (3) estimating the monetary value of the benefits accrued to each socio-economic group through multiplying the utilization rates by unit costs of relevant services; and, (4) summing total benefits within socio-economic groups resulting in total benefits for each quintile.

Individuals are categorised by income, proxied by equivalised per capita consumption, to calculate the value of the health sector subsidy received by each individual. The cost of provision of the various health services subsidized by UNHCR for consultations, drugs, diagnostics tests (labs and imaging, etc.), and hospitalisation, besides the running cost of the facilities, were collected from the 2017 UNHCR end of year financial report to estimate the unit cost and assess the benefits received by different groups. The unit cost is then calculated as the average cost by dividing total UNHCR expenditure in the specific service by total units used, taking into account that the net subsidy is weighted by the utilization rate to get the subsidy benefit of the individual.

The service-specific UNHCR subsidy received by an individual is,

$$S_{ki} = q_{ki} c_{k} - f_{ki} ,$$

where $q_{ki}$ indicates the quantity of service k utilized by individual i, ${c}_{k}$ represents the unit cost of providing k and $f_{ki}$ represents the amount paid for k by individual i.

The total UNHCR subsidy received by an individual is:

$$S_{i} = \sum\limits_{k} {\alpha_{k} \left( {q_{ki} c_{k} - f_{ki} } \right)}$$

where ${\alpha }_{k}$ are scaling factors that standardize utilization reference periods across services for the greatest share of the subsidy. We standardized on the reference period as for inpatient care reported over 1 year, then ${\alpha }_{k}$  = 1 for inpatient care and ${\alpha }_{k}$  = 13 for outpatient services as utilization was reported over only 4 weeks.

Given the difference between official and reported user payments for healthcare use, we experiment with two measures of the UNHCR benefit received as in similar papers in the literature [64]. The choice of measure directly affects the computation of the subsidy since it modifies the difference between the unit cost of care and fees paid by the individual. The “Benefits received” were then defined in this paper as either net benefits (NB) or gross benefits (GB).

Net benefits (NB)—are calculated as the cost of each service use, net of user fees and other related out-of-pocket expenditures (OOP), this is the net benefit received by the Population. While Gross benefits (GB)—are calculated as the total cost of each service type for the provider independently of the financing sources, this is the benefit allocated by UNHCR. Providers include non-governmental organisations (NGOs) which are partners of UNHCR and public hospitals and hospitals supported by UNHCR. Then, the GB represents the cost for the UNHCR in the absence of user fees and any other OOP, but with the same level of healthcare use. OOP was collected for each service during the survey, and mostly involved the cost contribution of refugees in the investigation tests and medications fees for outpatient healthcare use, as OOP expenditures for inpatient care were often fully covered by UNHCR. For those individuals who self-reported OOP expenditures higher than the value of the GB of the services, a zero value replaced the negative value following usual practice in previous papers [65].

CIs were calculated for healthcare utilization of both benefit measures, gross and net benefits. Also, CIs ranging between 0 and 1 indicated pro-rich distributions, while CIs ranging between 0 and − 1 indicated pro-poor distributions [27].

## Results

### Descriptive statistics

Table 1 shows the descriptive demographic characteristics for the 1872 individuals and 507 households included in the study.

**Table 1:** Household demographic characteristics (N = 1872 Individuals, 507 Households)

	No of observations/percentage	Mean ± SD
Age		24.5 ± 17.35
0–5 years	177 (9.5%)	
6–17	604 (32.3%)	
18–34	591 (31.6%)	
35–64	462 (24.7%)	
Above 65	38 (2.0%)	
Gender		
Male	973 (52%)	
Female	899 (48%)	
Persons with disability	64 (3.4%)	
Persons with chronic disease	381 (20.4%)	
Urban		
Rural	210 (11.2%)	
Urban	1662 (88.8%)	
Education of household head		
None, primary, or preparatory	1290 (68.9%)	
Secondary, Technical Institute	359 (19.2%)	
University or higher	223 (11.9%)	
Employment (household heads)		
Employed	262 (52.6%)	3.6 ± 2.2
Unemployed	245 (47.4%)	
Duration in the country		
< 2 years	347 (21%)	
Between 2 and 4 years	126 (7%)	
Between 4 and 5 years	1312 (67%)	
> 5 years	87 (5%)	
Governorates		
6 October and Giza	533 (28.5%)	
CAIRO	385 (20.6%)	
Alexandria	218 (11.6%)	
Qalyubia	217 (11.6%)	
Sharkia	125 (6.7%)	
Damietta	184 (9.8%)	
Others	210 (11.2%)	
Equivalized per capita consumption (quintiles)	$	
Lowest quintile	40.3	96 ± 58.1
2	69.8	
3	85.8	
4	109.1	
Highest quintile	182.4	

Table 2 reports the percentages of healthcare use by independent variables (unadjusted). Healthcare use is defined as a binary variable. Results for the Pearson’s chi-square test have been reported in this table comparing groups for the same categorical healthcare use.

**Table 2:** Percentages of healthcare use by independent variables (unadjusted)

	Outpatient visit, % (mean ± SD)			Inpatient admission, % (mean ± SD)		
	All	Public	Private	All	Public	Private
Total	0.31 ± 0.010	0.13 ± 0.008	0.18 ± 0.009	0.09 ± 0.007	0.07 ± 0.006	0.02 ± 0.003
Age						
0–5 years	0.24 ± 0.03	0.09 ± 0.02	0.15 ± 0.03	0.03 ± 0.01	0.01 ± 0.01	0.02 ± 0.01
6–17 years	0.16 ± 0.02	0.06 ± 0.01	0.10 ± 0.01	0.03 ± 0.01	0.02 ± 0.01	0.01 ± 0.00
18–34 years	0.25 ± 0.02	0.10 ± 0.01	0.15 ± 0.02	0.16 ± 0.02***	0.13 ± 0.01***	0.03 ± 0.01**
35–64 years	0.48 ± 0.02***	0.22 ± 0.02***	0.27 ± 0.02***	0.09 ± 0.01	0.08 ± 0.01	0.01 ± 0.00
> 65 years	0.84 ± 0.06***	0.46 ± 0.08***	0.38 ± 0.08***	0.18 ± 0.06*	0.13 ± 0.05	0.05 ± 0.04
Gender						
Female	0.34 ± 0.02***	0.13 ± 0.01**	0.21 ± 0.01**	0.13 ± 0.01	0.10 ± 0.01	0.03 ± 0.01
Male	0.25 ± 0.01	0.11 ± 0.01	0.14 ± 0.01	0.06 ± 0.01	0.04 ± 0.01	0.01 ± 0.00
Self/reported diseases						
Disability	0.36 ± 0.06	0.16 ± 0.05	0.20 ± 0.05	0.09 ± 0.04	0.05 ± 0.03	0.05 ± 0.03
NCD	0.75 ± 0.02***	0.33 ± 0.02***	0.42 ± 0.03***	0.14 ± 0.02**	0.11 ± 0.02**	0.03 ± 0.01
Residence						
Rural	0.33 ± 0.03	0.14 ± 0.02	0.19 ± 0.03	0.11 ± 0.02	0.09 ± 0.02	0.02 ± 0.01
Urban	0.28 ± 0.01	0.12 ± 0.01	0.17 ± 0.01	0.09 ± 0.01	0.07 ± 0.01	0.02 ± 0.00
Employment						
Unemployed	0.33 ± 0.02***	0.16 ± 0.01***	0.17 ± 0.01	0.09 ± 0.01	0.08 ± 0.01	0.01 ± 0.00
Employed	0.25 ± 0.01	0.08 ± 0.01	0.17 ± 0.01	0.09 ± 0.01	0.07 ± 0.01	0.03 ± 0.01*
Living standards—equivalized per capita consumption (quintiles)						
Poorest	0.23 ± 0.02	0.15 ± 0.02	0.08 ± 0.01	0.08 ± 0.01	0.06 ± 0.01	0.02 ± 0.01
2	0.29 ± 0.02	0.13 ± 0.02	0.16 ± 0.02	0.09 ± 0.01	0.07 ± 0.01	0.02 ± 0.01
3	0.31 ± 0.02	0.12 ± 0.02	0. ± 0.02	0.10 ± 0.02	0.07 ± 0.01	0.03 ± 0.01
4	0.28 ± 0.02	0.10 ± 0.02	0.18 ± 0.02	0.09 ± 0.01	0.08 ± 0.01	0.01 ± 0.01
Richest	0.36 ± 0.02*	0.11 ± 0.02	0.25 ± 0.02***	0.10 ± 0.02	0.07 ± 0.01	0.02 ± 0.01

Pearson’s chi-square test

*p < 0.05, **p < 0.01, ***p < 0.001

Regarding the outpatient services use, 31% of the individuals declared to have used the services over the previous month period. On the contrary, only 9% had used inpatient services over the preceding 12-month period. Refugees used private health facilities more than public ones for outpatient care (18% vs. 13%), while public hospitals including UNHCR supported facilities were more frequently used for inpatients care (public health facilities more for inpatient care (7% vs. 2%). Total (either public or private) outpatient healthcare use was significantly higher (*p* < 0.001) for individuals ages between 35 and 64 years also for those above 65 years, female individuals with NCD, those unemployed, and those in the richest quintile (*p* < 0.05). In the case of hospital use, total (either public or private) use was significantly higher (*p* < 0.05 or lower) for those between 18 and 34 years, those above 65 years, and individuals with NCD.

The decomposition analysis for total healthcare utilization which includes both public and private use for each outpatient and inpatient service is shown in Table 3, as well the contribution of each covariate to the overall healthcare inequality.

**Table 3:** Socio-economic inequality decomposition in the probability of healthcare utilization by need, consumption, and other non-need factors

Variables	Concentration index of covariates (C_*i*,*K*_)	Total healthcare utilization			
		Outpatient services		Inpatient services	
		Elasticity (b)	Contribution towards inequality (C_*i*,*K*_) * b)/CI	Elasticity (b)	Contribution towards inequality (C_*i*,*K*_) * b)/CI
Need factors					
Age	0.0180	0.0645	1%	0.2524	23.1%**
Gender (male)	0.0067	− 0.1492	− 1%***	− 0.4497	− 15.4%***
Disability^b^	0.0735	0.0014	0.2%	0.0037	1%
NCD	− 0.0146	0.4059	− 6.3%***	0.0735	− 5.5%**
Non-need factors					
Urban	0.0086	− 0.2478	− 2.3%*	− 0.0092	− 0.4%
Governorates (Greater-Cairo)	− 0.0248	− 0.0295	1%	0.1993	− 25%
Household size	− 0.0221	− 0.2479	6%**	− 0.2997	33.7%*
Education (Head of household)	0.0207	0.0488	1.1%	− 0.1917	− 20%
Duration in the country	− 0.0009	0.2020	− 0.2%*	0.6526	− 3%***
Knowledge	− 0.0385	0.0148	− 0.6%	− 0.0204	4%
Employment (Head of household)	0.0038	− 0.0309	− 0.1%	0.1233	2.4%
Per-capita consumption	0.2874	0.1799	54.4%***	0.0128	18.7%
Wagstaff’s Index (CI_W_)	**0.095**			**0.02**	
*Erreygers’ Index (CI*_*E*_*)*	*0.079*			*0.006*	

Bold is used to highlight the main findings and used in the discussion

Italics used for another correction of the *CI*, added for comparaison

*CI* concentration index; CIs are Wagstaff normalized indices. Erreygers’ Index = (4μ/b − a) * CI, where, a and b are upper and lower limits of the health variable, CI is the standard concentration index, and μ is the mean of the health variable. CI_E_ = 4μ(1 − μ)CI_W_

^b^Elasticity (b)=(β_i_^m^X_j_/μ) , indicates the impact of each determinant on the desired health outcome

^*^p < 0.05, **p < 0.01, ***p < 0.001

Each contribution is the product of the sensitivity of health with respect to the corresponding determinant and the degree of consumption-related inequality in that determinant. If the contribution of a factor is positive, this means that the utilization inequality would have been lower if that factor was not present (the opposite for negative contribution). From the need factors, NCDs was found more common among individuals from poorer households (negative CI) while the use of outpatient and inpatient services is higher among those with NCDs (positive elasticity), this has negatively contributed with 6.3% and 5.5% respectively, to the total inequality in outpatient and inpatient utilisation. Moreover, males are slightly more concentrated among richer households (positive CI), but the use of both services is higher among females (negative elasticity), contributing to the pro-poor inequality in the total inequality in each service by 1% and -15.4%. While age positively contributed by 23% to total inequality in the inpatient service use which cancelled out the pro-poor distribution in gender and NCD in the need factors.

In the non-need factors, poor refugees were found concentrated more in rural areas, big families and with longer duration in the country. Refugees who have been longer in the country use significantly both health services. While The household size contributed to the pro-rich utilization in both outpatient and inpatient services, by 6% and 33.7% respectively. The contribution of the living standard proxied by the consumption has significantly contributed to the total inequality in outpatient and inpatient services with 54.4% and 18.7%, respectively.

### Concentration indexes and horizontal inequity

Table 4 shows the probability of healthcare utilization across living standards quintiles, Wagstaff normalized indices (CI), and horizontal inequity (HI) for the probability of public, private and total outpatient visits and inpatient utilization.

**Table 4:** Quintile distribution, inequality, and inequity in healthcare utilization

	Public outpatient visits	Private outpatient visits	Public inpatient admission	Private inpatient admission	Total outpatient visits	Total inpatient admission
Quintiles						
Lowest quintile	0.153	0.086	0.061	0.018	0.225	0.079
2	0.137	0.163	0.069	0.022	0.280	0.091
3	0.120	0.200	0.077	0.026	0.310	0.103
4	0.116	0.186	0.068	0.010	0.276	0.078
Highest quintile	0.116	0.257	0.073	0.020	0.336	0.094
CI	− 0.073	0.214	0.022	0.014	0.095	0.02
*Cneed*_*predicted*	− 0.002	− 0.011	0.001	0.000	− 0.008	0.001
HI (inequity)	− 0.072*	0.224***	0.02	0.014	0.104***	0.02

*CI* concentration index, CIs are Wagstaff normalized indices. *HI* horizontal inequity index, *C*_*need*_*predicted*_ concentration index of need-predicted use

^*^p < 0.05, **p < 0.01, ***p < 0.001

The CIs for the probability of utilization of all services were positive, indicating that healthcare use was pro-rich, except for public outpatient use (CI = − 0.073), which is pro-poor, indicating that the worse-off living standards group use public outpatient services more than the better-off group. The mean probability of use of the poorest in the utilization of public outpatient care was 15.3% during the previous month, a number that decreases monotonically with consumption to 11.6% for the highest two quintiles. The utilization of private outpatient services was higher for the richest quintile (25.7%) than for the poorest one (8.6%).

The CIs for the probability of private outpatient visits (CI = 0.214) and both public and private inpatient admission CIs (CI = 0.02; 0.014 respectively) were all positive, which means that the richest had more advantages than the poorest in the probability of access to those healthcare services. Overall, poorer population groups have greater healthcare needs while richer ones use the services more extensively with CI = 0.095 of total outpatient services and CI = 0.02 of total inpatient services, and the pro-rich horizontal inequity is mainly engrained in socioeconomic inequalities.

After controlling for need factors, we have obtained the horizontal inequity index (HI). The need-predicted distribution was pro-poor in the probability of use for all outpatient services. This is because ‘need’, as proxied by demographics and NCDs, was more concentrated among the lower socioeconomic groups.

HIs for the probability of using public outpatient visits showed pro-poor indices (− 0.072) but it was observed a pro-rich inequity for private outpatient use (CI: 0.224), as well as for both types of inpatient services (0.02; 0.014). HI indices for the probability of total healthcare use for outpatient and inpatient services were also both positive (0.104; 0.02).

### Healthcare subsidies

Inpatient services consume 71.4% of the total subsidies of UNHCR versus 28.6% allocated to outpatient services.

Table 5 shows the average net and gross benefit of each quintile for both outpatient and inpatient subsidised health services and the concentration index of BIA for both subsidies.

**Table 5:** Utilization percentages of subsidies by consumption quintiles and concentration index for benefit incidence

	Subsidy for outpatient services		Subsidy for inpatient services	
Percentage of total subsidies	28.6%		71.4%	
Per capita consumption	Net benefit	Gross benefit	Net benefit	Gross benefit
Lowest quintile	9.5	23.8	12.5	18.7
2	10.2	21.2	9.4	22.4
3	15.6	18.5	21.5	26.9
4	18.9	18.3	47.6	10.4
Highest quintile	45.9	18.2	9.0	21.5
**Concentration index (CI)**	0.3723***	− 0.0643*	**0.1023**	**0.0134**

^a^Net benefit (proportional cost assumption)

^b^Gross benefit (linear cost assumption relative to utilization)

Inpatient services consume 71.4% of the total subsidies of UNHCR versus 28.6% allocated to outpatient services.

The share of gross benefits (GB) for outpatient care is higher for the first two quintiles (23.8% and 21.2%, respectively) than for the remaining three quintiles (around 18%). Then, the estimated CI is significant and negative indicating that outpatient GB distribution is pro-poor. The share of GB for inpatient care does not show a specific trend in distribution among consumption quintiles. The highest, second and third quintiles show a higher proportion of the GB than the fourth and lowest quintiles. The CI for GB inpatient benefits is positive, indicating a pro-rich distribution, but it is not statistically significant.

The proportion of net benefits (NB) in Table 5, tends to increase with consumption for outpatient services and is higher for the highest quintiles. The richest 20% of the households received 45.9% of NB from outpatient care, and the richest 40% received nearly two thirds (64.8%) of those total NB. The proportion of NB from inpatient care is highly concentrated in quintiles 3 and 4 (69.1%), but the proportion of NB accruing to the richest quintile (9%) is lower than in the case of outpatient services. Both CIs for the net benefit from outpatient and inpatient care are positive indicating a pro-rich distribution, being higher and statistically significant for the CI in the case of outpatient care.

There are some important changes between the distribution of gross and net benefits from UNHCR subsidies both for outpatient and inpatient services use. These differences indicate that OOP expenditures are not distributed proportionally to healthcare use among consumption quintiles. Although results in Table 5 do not show a clear pattern, they indicate that the burden of OOP expenditures for the poor contributed to increasing the share of NB accruing to the highest consumption quintiles, except for the highest quintile for inpatient use. As a result, CIs for net benefits from UNHCR are pro-rich.

As reported in previous literature [65], the bias in self-declared OOP expenditures, especially truncation at zero for net benefits, which mutes differences and modifies aggregate net benefits, and the number of observations with positive net benefits in each quintile may greatly influence the magnitude of changes in the share of NB compared to GB and converting benefit shares for GB and NB not comparable. Further analysis will be needed to test the significance and stability of these differences and to test for explanatory behaviours.

## Discussion

To our knowledge, this is the first study to estimate and decompose inequalities in healthcare use among refugees and evaluate the distribution of resources that enable healthcare access. Two previous papers have described healthcare access and utilization among Syrian refugees in Jordan and Canada [69, 70], however, inequality decomposition and estimation of concentration indices for use and benefit distribution were not performed.

The main three results obtained in this study, for Syrian refugees in Egypt, may be summarised as follows; First, we found pro-rich inequality and horizontal inequity in the probability of refugee’s outpatient and inpatient total health services utilization; overall, poorer population groups were found to have greater healthcare needs while richer groups use health services more extensively. Whereas some studies in low and middle-income countries have shown that the poorest population groups tend to use primary care more extensively than the rich population [71, 72], other studies have similarly found that need factors such as age, gender, and self-reported health status operate in a pro-poor direction of healthcare use [45, 73–75].

Second, decomposition analysis showed that the main contributor to inequality is socioeconomic status, with other elements such as large families, being a female, the presence of chronic disease and duration of asylum in Egypt further contributing to inequality. These findings call attention to patients with chronic illnesses, who are more concentrated among the poor and presumably have greater healthcare needs, seek outpatient visits more frequently, and are more likely to need hospital admissions. These patients should be considered by UNHCR among the most vulnerable groups in need of social protection. Also, refugees with longer stays in the country have a significant effect toward the pro-poor effect, probably due to the depletion of their savings. Nevertheless, the longer refugees stay in the country, the better they become accustomed to the utilization of the system. While high-income groups are likely to have relatively good access to health services, further analysis may be needed to explore the health-seeking behaviours of the refugees. Although inpatient need is high and should affect the utilization of services, our results point out that fees associated with inpatient care in Egypt may represent a barrier that results in inequities.

Third, the BIA showed that the net benefit distribution of subsidies of UNHCR for outpatient and inpatient care is also pro-rich after accounting for out-of-pocket expenditures. Some studies [38, 76] explain how these results might be linked to the fact that the poor cannot afford to be ill, be it because of the opportunity cost of lost work time, the lack of knowledge, or in general to poor health service access. Measurement of the net benefits shows that the higher income groups benefit from the highest share of UNHCR subsidies since they have some means to bear the brunt of the direct and indirect cost of the services (user fees and OOP) whatever is the service provider, while the lowest income group does not receive a proportionate share of net benefits, which means that the allocation is poorly targeted. This finding is in line with another study focusing on several countries in Africa [75] and with some recent studies focusing on outpatient net benefits in China [62], India [63, 65]. The inpatient NB observations may not be surprising as it is generally believed that spending on hospitals primarily benefits the rich [77, 78] while the poor’s access is limited for a variety of reasons—for instance, user fees and location of hospitals and the fact that hospitals tend to be more specialized and offer services not aimed at curing the common ills of the poor [79]. Our benefit incidence analysis results may be interpreted in the sense that user-fees and out of pocket expenditure represent an additional barrier to the access of health in both outpatient and inpatient services for the poor population.

To ensure higher benefits for the poor from spending, UNHCR needs to adopt policies that encourage the poor to utilize primary health services more intensively than the non-poor, while other modalities for services delivery as proportional cost reimbursement or cash-based intervention should be looked at with caution, as they may have implications that endanger the poorest groups fair access to healthcare.

Thus, BIA could inform policymakers on how well spending on a health service is targeted, (e.g., primary healthcare vs. secondary and specialised healthcare), how it compares with the incidence of other types of needs.

Finally, this study has some limitations and potential methodologic issues that should be carefully considered for generalisation of the results and further research.

First, this analysis stalks from the self-reported nature of the data, the problem of recall period bias as well as the subjective measurement of healthcare use and health status are limitations of using survey data in empirical studies, this may have led to possible over-estimation of variations in utilization and measurement error in our sample [80, 81].

Furthermore, in our model specification and estimation, there is potential bias as a result of omitted variables that were not included in the explanatory variables. While we did our best to specify the model that included all necessary variables based on operational and empirical evidence, no model can ever be truly “complete” because of potential omitted variables not known to the researchers at the time of analysis, or due to data limitations.

Second, several criticisms have been raised in recent literature about the decomposition technique [82, 83], and have mainly focused on refining the technique to get more precision on the estimation of HI and to better explain the sources of inequity in healthcare use [84]. Other criticisms concerned the possibility to, only, correctly decompose one form of rank dependent index, while there are several rank dependent measures used in the literature [82, 83]. Furthermore, the conventional method of measuring socioeconomic inequity based on needs-adjusted utilization may not necessarily imply inequity because these differences may be explained in part by individuals’ informed choices and preferences [85]. Second, distribution of needs-adjusted utilization by socioeconomic status may not be equitable if the services being used are of low quality or are inappropriate [86]. Finally, one of the main limitations of this approach to measuring inequity is that it does not offer a causal interpretation of the findings [83].

Third, a major limitation of BIA methodology [87] is that subsidy per unit of usage may not be the best indicator of benefits as it is unlikely to reveal the real value (similarly with the marginal rates of substitution of private goods) that consumers attach to that good. Also, self-declared OOP expenditures may be biased and the truncation to zero of negative net benefits adopted and justified in the literature when the amount paid OOP exceeds the cost of the services [64, 65] may influence net benefit distribution.

In addition, because we used cross-sectional data for our analysis, there could be reverse causality exist between health care utilization and out-of-pocket payment; hence one must be cautious in interpreting this result. Understanding the implications of financial barriers such as the role of co-payments for the observed pro-rich inequity would be useful for policy objectives.

## Conclusion

This paper sheds light on the challenges and opportunities of refugees’ access to healthcare and uncover sources of inequities that require further attention and advocacy by policymakers. Our results showed that without equitable subsidy and efficient allocation, poor refugees could not afford healthcare services. We found that the richest had more advantages than the poorest in the probability of access to healthcare services and that the main contributor to total inequality is the socioeconomic status, with other elements such as large families, the presence of chronic disease, being female and the duration of asylum in Egypt further contributing to inequality. Besides, the burden of OOP expenditures on the poor contributed to increasing the share of net benefits accruing to the highest income quintiles, and consequently to the surge of inequality of the use of subsidised services.

Our findings can be interpreted in favour of implementing an equity lens on the inpatient and secondary healthcare program of UNHCR, to mitigate the service skew towards the well-off, and to ensure key needs are met without leaving the most vulnerable behind.

In a context in which refugees’ integration in the national system is a key strategic plan for UNHCR, improving the management and quality of primary public services will further encourage the utilization of public facilities among the refugees and offer a cost-efficient solution and relative financial protection for lower-income refugees who manifest higher-need. Although the Government of Egypt endorsed a National Health Insurance law in 2018, this scheme is foreseen to be implemented over several years, refugees have not been given access yet. Until then, the financial protection of refugees depends exclusively on the subsidiary schemes offered by UNHCR.

Ultimately, a health policy alone is not enough to tackle issues of inequality, a comprehensive social policy that encompasses education and employment opportunities for refugees, as well as pro-poor welfare, is needed.

## Data Availability

The datasets used and/or analysed during the current study are available from the corresponding author on reasonable request.

## Abbreviations

ANC: Antenatal care
BIA: Benefit incidence analysis
CI: Concentration index
GB: Gross benefit
HI: Horizontal inequity
NB: Net benefit
NCD: Non-communicable chronic disease
NGO: Non-Governmental Organization
OOP: Out-of-pocket expenditures
UNHCR: United Nations High Commissioner for Refugees
UHC: Universal Health Coverage (UHC)
